# Iron Deficiency in CKD Without Concomitant Anemia

**DOI:** 10.1016/j.ekir.2021.07.032

**Published:** 2021-08-10

**Authors:** Jay B. Wish, Stefan D. Anker, Javed Butler, Aleix Cases, Austin G. Stack, Iain C. Macdougall

**Affiliations:** 1 Division of Nephrology, Indiana University Health, Indianapolis, Indiana, USA; 2 Department of Cardiology (CVK), Charité Universitätsmedizin, Berlin, Germany; 3 Berlin Institute of Health Center for Regenerative Therapies (BCRT), Charité Universitätsmedizin, Berlin, Germany; 4 German Centre for Cardiovascular Research (DZHK) Partner Site Berlin, Charité Universitätsmedizin, Berlin, Germany; 5 Department of Medicine, University of Mississippi School of Medicine, Jackson, Mississippi, USA; 6 Institut d’Investigacions Biomèdiques August Pi i Sunyer (IDIBAPS), Universitat de Barcelona, Barcelona, Spain; 7 Department of Nephrology, University Hospital Limerick and School of Medicine, University of Limerick, Limerick, Ireland; 8 Department of Renal Medicine, King’s College Hospital, London, UK

**Keywords:** anemia, chronic kidney disease, heart failure, iron deficiency

## Abstract

The physiological role of iron extends well beyond hematopoiesis. Likewise, the pathophysiological effects of iron deficiency (ID) extend beyond anemia. Although inextricably interrelated, ID and anemia of chronic kidney disease (CKD) are distinct clinical entities. For more than 3 decades, however, nephrologists have focused primarily on the correction of anemia. The achievement of target hemoglobin (Hgb) concentrations is prioritized over repletion of iron stores, and iron status is generally a secondary consideration only assessed in those patients with anemia. Historically, the correction of ID independent of anemia has not been a primary focus in the management of CKD. In contrast, ID is a key therapeutic target in the setting of heart failure (HF) with reduced ejection fraction (HFrEF); correction of ID in this population improves functional status and quality of life and may improve cardiovascular (CV) outcomes. Given the strong interrelationships between HF and CKD, it is reasonable to consider whether iron therapy alone may benefit those with CKD and evidence of ID irrespective of Hgb concentration. In this review, we differentiate anemia from ID by considering both epidemiologic and pathophysiological perspectives and by reviewing the evidence linking correction of ID to outcomes in patients with HF and/or CKD. Furthermore, we discuss existing gaps in evidence and provide proposals for future research and practical considerations for clinicians.

The association between CKD and anemia has been recognized for nearly 2 centuries.^1^^,^^2^ Contemporary research has revealed that anemia of CKD is independently associated with a range of adverse outcomes, including reduced quality of life, hospitalization, progression to kidney failure, major CV events, and death.^3^, ^4^, ^5^, ^6^, ^7^ The burden of CKD anemia increases with declining kidney function. International studies reveal that the prevalence of anemia—defined as a Hgb concentration <12.0 g/dl—is substantial, at 30%, 46%, and 72% in patients with stage 3b CKD, stage 4 CKD, and stage 5 CKD without kidney replacement therapy, respectively.^8^ Moreover, nearly 85% of patients with stage 5 CKD treated with hemodialysis in the United States have Hgb levels <12.0 g/dl.^9^

ID, whether resulting from a lack of iron stores (absolute ID) or the inability to use existing iron stores (functional ID), is a major contributor to the development of anemia in patients with CKD.^2^^,^^7^ ID, however, is not the sole cause of CKD anemia; other contributory mechanisms include relative erythropoietin deficiency and the shortened life span of red blood cells.^2^^,^^7^ Naturally, patients with CKD can also experience non–CKD-associated causes of ID and anemia, including infection, gastrointestinal bleeding, and hemoglobinopathies.

Many patients with CKD meet criteria for ID without clinical evidence of anemia.^8^^,^^10^, ^11^, ^12^, ^13^ For more than 3 decades, nephrologists have focused primarily on the correction of anemia in patients with CKD using erythropoiesis-stimulating agents (ESAs) and supplemental iron. Historically, the main objective of therapy has been achievement of a specific Hgb target, based on the premise that this alone is the principal determinant of clinical outcomes. The correction of iron stores and functional iron reserves ensuring an adequate supply of iron to the bone marrow has traditionally been viewed as a secondary objective, and one that is only targeted in patients with evidence of anemia.

A central question surrounding the management of anemia in CKD posed in this review is whether ID should be considered, and treated, as an entity distinct from anemia defined by Hgb targets. The assertion that ID *per se* is a distinct entity and should be treated independently of anemia should be supported by strong biological plausibility, epidemiologic data, and robust evidence from clinical trials.^14^, ^15^, ^16^, ^17^ The use of iron supplementation by nephrologists is quite different from the approach used by cardiologists in the setting of HF. In cardiology, iron is administered with the primary goal of correcting ID, improving functional status and quality of life, and potentially reducing CV events (notably hospitalizations for HF), not correcting anemia and increasing Hgb levels.^18^, ^19^, ^20^, ^21^ In contrast, in nephrology, management of ID is primarily a means to correct anemia of CKD and to achieve a specific Hgb target. Thus, the correction of ID independent of anemia is not a primary consideration. By limiting the use of iron to patients with CKD with evidence of anemia and ID, it is possible that nephrologists are excluding a relatively large population of patients who would otherwise benefit from the therapy.

This review will evaluate whether nephrologists should consider iron therapy for patients with CKD and evidence of ID irrespective of Hgb concentration. Such an approach could be contrasted with current guidelines that “only” recommend consideration of iron therapy for adults with anemia (defined as Hgb <13.0 g/dl in men and <12.0 g/dl in women) and evidence of ID.^7^ We will evaluate the differences between anemia and ID from epidemiologic and pathophysiological perspectives before reviewing the current evidence from published clinical trials in HF and CKD. We will further explore gaps in our knowledge before concluding with recommendations for future research and practical considerations for clinicians.

### Epidemiology of Anemia Versus ID

Although this review aims to evaluate the implications of considering, for the purposes of management, ID as a clinical entity distinct from anemia of CKD, we acknowledge that ID is a key contributor to the anemia of CKD, and thus both are inextricably interrelated. Depletion of iron stores will, if left untreated, progress to impaired erythropoiesis and reduced Hgb concentrations.^22^ In the setting of CKD, ID results from blood loss, reduced iron intake/absorption, and reduced mobilization of iron from storage cells secondary to increased hepcidin concentrations.^23^

There are limited data on the concurrent prevalence of ID and anemia within the same population of patients with CKD. In a cohort of 700 kidney transplant recipients with a mean (SD) estimated glomerular filtration rate (eGFR) of 52.3 (20.2) ml/min per 1.73 m^2^, 30% of the patients had ID (transferrin saturation [TSAT] <20% and ferritin <300 ng/ml) without anemia (World Health Organization criteria: Hgb <13.0 g/dl for men and <12.0 g/dl for women) and 34% had anemia without ID.^24^ Wong *et al.*^8^ found the prevalence of ID among patients with CKD varied across countries, but not appreciably across stages of CKD. In contrast, the prevalence of anemia increased substantially with advancing stage of CKD. The lack of additional data on ID among patients with CKD is not unexpected, given that assessment of iron parameters (i.e., TSAT and ferritin) is generally prompted by the presence of anemia, and typically restricted to patients with CKD and anemia.^7^ Even when indicated, real-world assessment of iron parameters occurs at suboptimal rates. For example, in the United States, only 47% of patients with CKD stages 3 to 5 and Hgb <10.0 g/dl had iron indices measured.^8^ Although impractical for routine assessment, bone marrow staining is the gold standard for assessment of iron status. In clinical practice, reliance on laboratory assessments—particularly, TSAT <20%—has been recommended for both HF and CKD populations.^25^^,^^26^

Data on the prevalence of ID in the setting of HF are more readily available than those observed for CKD. Reduced intracellular iron in the setting of HF is thought to result from inhibition of transferrin receptor protein 1 secondary to a combination of systemic inflammation, overactivity of the renin-angiotensin-aldosterone system, and increased sympathetic nervous activity.^27^ As reviewed by Rocha *et al.*,^27^ the prevalence of ID was 30% to 50% among patients with stable HF and between 50% and 80% among patients with acute HF. In an international cohort of >1500 patients with HF, the prevalence of ID, CKD, and anemia was 50%, 28%, and 28%, respectively.^11^^,^^28^ Among those patients with CKD and ID (in addition to HF), 58% did not have concomitant anemia. Conversely, among patients with CKD and anemia (in addition to HF), 38% did not have concomitant ID. The burden of ID generally increases with worsening HF (as determined by the New York Heart Association classification).^28^^,^^29^ ID is a common comorbidity of HF regardless of whether left ventricular ejection fraction is reduced or preserved.

### Distinguishing the Clinical Effects of ID Versus Anemia

#### Heart Failure

Given that iron is necessary for multiple cellular processes, including energy production, DNA synthesis, drug metabolism, steroid synthesis, immune function, drug metabolism, and oxygen transport,^30^^,^^31^ it is not surprising that the effects of ID are wide ranging. These effects can be broadly classified as those resulting from either hematopoietic or nonhematopoietic pathway (Figure 1).^32^ From a temporal perspective, impairment in muscle function, immunologic defense, neuronal functioning, and exercise tolerance seems to occur long before reductions in Hgb concentrations.^22^ In the setting of HF, dysfunctional mitochondrial energy production (a potential consequence of ID) in the heart, skeletal muscle, and kidneys is associated with reduced systolic function, exercise intolerance and fatigue, and kidney impairment, respectively.^33^, ^34^, ^35^ The impact of mitochondrial dysfunction on the heart, skeletal muscle, and kidneys is strongly aligned with the high metabolic demands and energy consumption of these organ systems and tissues.^36^

**Figure 1:**
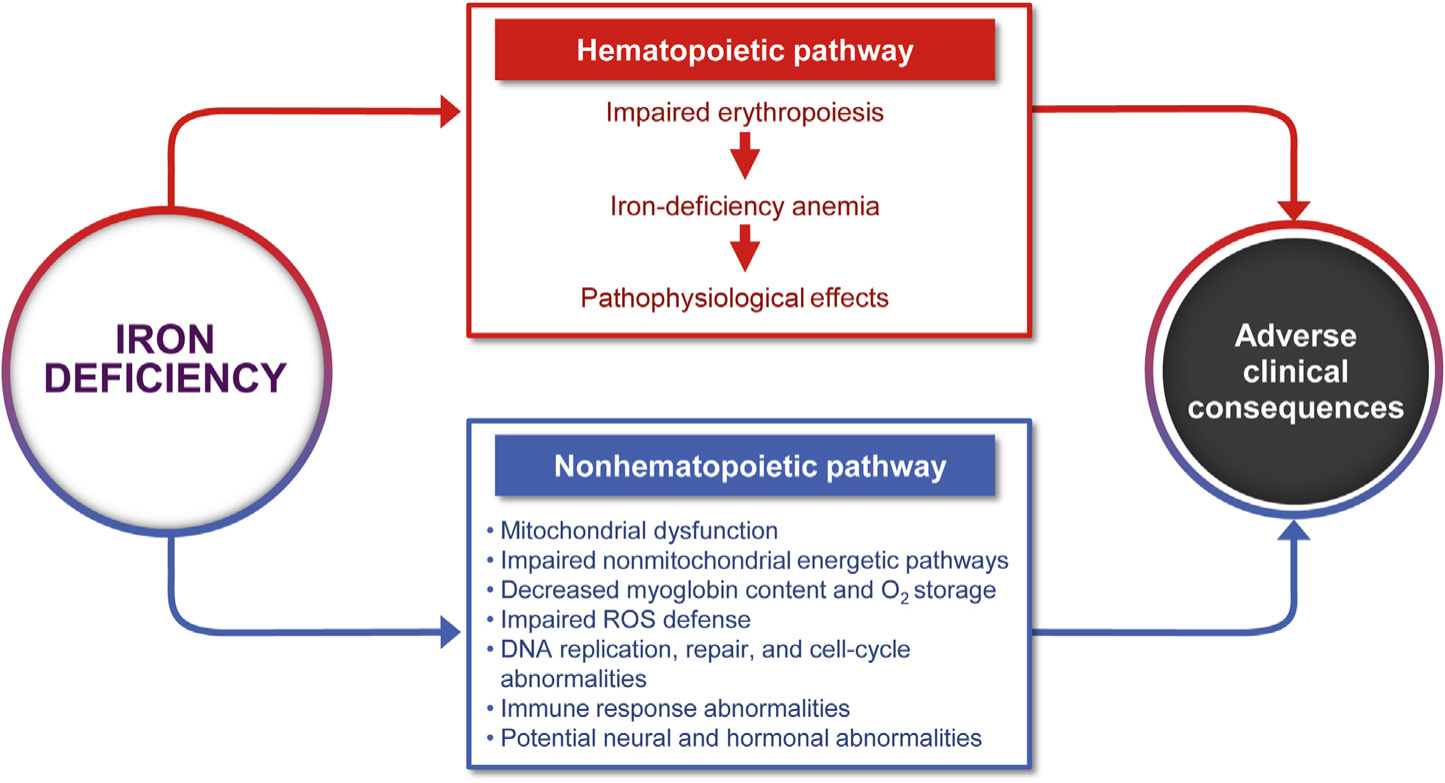
Pathophysiological consequences of iron deficiency. Adapted from Ponikowski *et al.*^32^ O_2_, oxygen; ROS, reactive oxygen species.

The impact of ID on functional outcomes in HF has been well documented. In a study of 443 patients with HFrEF, ID was an independent predictor of reduced exercise capacity as assessed by peak oxygen consumption and ventilatory response to exercise.^37^ The impact of ID alone (i.e., in the absence of anemia) was greater than that observed with anemia alone (i.e., in the absence of ID). Similarly, Comín-Colet *et al.*^38^ found that ID, and not anemia, was independently associated with reduced health-related quality of life among patients with HF. Observed declines in quality of life were largely driven by reductions in physical domain components (e.g., walking or climbing stairs, appetite, fatigue). The observed impact of ID on quality of life is substantial and has been replicated by other investigators.^39^

Several studies have identified strong independent associations of ID with mortality in patients with HF. Klip *et al.*^11^ reported a 42% increased risk of death for patients with ID in a cohort study of 1506 patients with HF. This association persisted regardless of CKD and/or anemia.^11^ Similarly, in a Belgian cohort, Martens *et al.*^29^ found that ID, in the absence of anemia, was also an independent risk factor for death and hospitalization in patients with HF. In contrast, they found that anemia, in the absence of ID, did not predict HF hospitalization and was a weaker predictor of mortality. Notably, the independent association between ID and adverse outcomes was not affected by left ventricular ejection fraction.

#### General Population and CKD

In contrast to HF—in which the presence of ID has independently been associated with impaired exercise tolerance^37^ and increased mortality^19^—there are limited outcome studies of ID in the absence of anemia in the general population and patients with CKD. In an analysis of data from almost 16,000 adults in the Third National Health and Nutrition Examination Survey (1988–1994), Stack *et al.*^40^ found that TSAT levels <23.7% were independently associated with increased CV and all-cause mortality (relative to the reference group; TSAT 23.7%–31.3%) after adjustment for multiple confounders, including kidney function and Hgb. Recent data from Germany also reveal that >50% of healthy adults without anemia met criteria for functional ID (i.e., ferritin ≤100 ng/ml or ferritin 100–299 ng/ml with TSAT <20%).^16^ In multivariate analyses adjusting for Hgb and CV risk factors, functional and absolute ID were associated with 30% and 90% increases in all-cause mortality, respectively, in a median follow-up of approximately 10 years.

In a large cohort of patients with CKD not requiring dialysis, functional ID was more strongly predictive of 1-year mortality than absolute ID (relative to a reference group of patients without ID) despite similar Hgb levels across all groups (mean ~12.0 g/dl).^41^ In the same cohort, Cho *et al.*^42^ revealed that patients with CKD and low iron (TSAT 0.4%–16% and ferritin 0.9–55 ng/ml) were significantly more likely to develop HF or die relative to those with TSAT 16% to 28% and ferritin 55 to 205 ng/ml. Patients in the high-iron group (TSAT 28.0%–99.5% and ferritin 205–4941 ng/ml) exhibited reduced rates of hospitalization for HF. Similarly, in an international cohort of patients with CKD not requiring dialysis (*N* = 5144), TSAT levels <25% independently predicted all-cause mortality after adjustment for Hgb levels.^43^ In a separate cohort of kidney transplant recipients, ID independently predicted all-cause mortality (hazard ratio [95% CI]: 1.74 [1.10–2.73]),^24^ whereas anemia failed to predict higher mortality after adjustment for ID, eGFR, and proteinuria. An analysis of approximately 2500 patients with CKD without kidney replacement therapy found that ID (as assessed by lower TSAT) was associated with significantly lower physical health-related quality of life after adjustment for Hgb levels.^44^

### Approach to Treatment

#### Correcting Anemia Versus ID in CKD

The presence of ID and/or anemia in patients with CKD should be fully evaluated, and correctable causes should be addressed whenever possible.^7^ When easily reversible causes are not identified, what should be the therapeutic target(s)? To date, clinical guidelines have focused primarily on the achievement of specific Hgb targets in patients with CKD so as to avoid the need for blood transfusions and to improve quality of life associated with severe anemia (i.e., <9.0 g/dl).^7^ Full correction of anemia with ESAs in patients with CKD, however, has been associated with major adverse clinical outcomes. Studies of ESAs in CKD populations (e.g., the Normal Hematocrit Study,^45^ the Correction of Hemoglobin and Outcomes in Renal Insufficiency study,^46^ the Cardiovascular Risk Reduction by Early Anemia Treatment with Epoetin Beta study,^47^ and the Trial to Reduce Cardiovascular Events with Aranesp Therapy^48^) have revealed unequivocally that targeting higher Hgb levels (i.e., Hgb ≥13.0 g/dl) is not associated with improved CV outcomes or survival but, in fact, is associated with harm.^49^ The current evidence base (Table 1) suggests that correction of anemia, at least with ESAs, does not optimize outcomes in patients with CKD.

**Table 1:** Trials evaluating the safety and efficacy of full correction of Hgb in patients with CKD and anemia

Study	Patient population	Treatment	Anemia status correction target	Results
Normal Hematocrit Study^45^	Patients with HF or IHD undergoing HD (*N* = 1233) NYHA classes II–III HF: ~69%	Epoetin	Hematocrit: 42% vs. 30%	Higher hematocrit was associated with an increased risk of death or MI^a^ Risk ratio (95% CI): 1.3 (0.9–1.9)
CHOIR^46^	Patients with CKD (eGFR of 15–50 ml/min per 1.73 m^2^) (*N* = 1432) HF: ~24%	Epoetin α	Hgb: 13.5 g/dl vs. 11.3 g/dl^b^	Higher hematocrit was associated with an increased risk of the composite of death, MI, hospitalization for HF, and stroke^c^ Hazard ratio (95% CI): 1.34 (1.03–1.74)
CREATE^47^	Patients with CKD (eGFR of 15–35 ml/min per 1.73 m^2^) (*N* = 603) HF: ~32%	Epoetin β	Hgb: 13.0–15.0 g/dl vs. 11.0–12.5 g/dl	Similar rates of first CV events between Hgb targets Hazard ratio (95% CI): 0.78 (0.53–1.14)
TREAT^48^	Patients with T2D and CKD (eGFR of 20–60 ml/min per 1.73 m^2^) (*N* = 4038) HF: ~33%	Darbepoetin alfa vs. placebo	Hgb: 13.0 g/dl vs. placebo^d^	Darbepoetin alfa was associated with a numerically increased risk of the composite of death, MI, HF, stroke, and hospitalization for myocardial ischemia Hazard ratio (95% CI): 1.05 (0.94–1.17)

CHOIR, Correction of Hemoglobin and Outcomes in Renal Insufficiency; CKD, chronic kidney disease; CREATE, Cardiovascular Risk Reduction by Early Anemia Treatment with Epoetin Beta study; CV, cardiovascular; eGFR, estimated glomerular filtration rate; HD, hemodialysis; HF, heart failure; Hgb, hemoglobin; IHD, ischemic heart disease; MI, myocardial infarction; NYHA, New York Heart Association; T2D, type 2 diabetes; TREAT, Trial to Reduce Cardiovascular Events with Aranesp Therapy.

a Study was terminated by the independent data monitoring committee after a median follow-up of 14 months.

b Before a protocol amendment, Hgb targets were 13.0–13.5 g/dl and 10.5–11.0 g/dl.

c Study was terminated by the independent data monitoring committee after a median follow-up of 16 months.

d Rescue darbepoetin alfa was administered if Hgb was <9.0 g/dl.

Kidney Disease: Improving Global Outcomes guidelines for the management of anemia in CKD were last published in 2012.^7^ At the time, iron supplementation for the treatment of ID in the context of anemia was recommended to raise Hgb levels and reduce ESA doses. More recently, evidence has emerged that a proactive approach to i.v. iron therapy is associated with clinical benefit among patients with CKD undergoing maintenance hemodialysis from the PIVOTAL (Proactive IV IrOn Therapy in HaemodiALysis Patients) trial. It is noteworthy that although all patients were receiving ESAs for the treatment of anemia, the mean baseline Hgb was 10.6 g/dl, with approximately 25% of patients having Hgb levels <9.6 g/dl and 25% of patients having levels >11.5 g/dl.^50^ Patients in PIVOTAL were randomized to either high-dose, proactively administered i.v. iron (400 mg monthly in the absence of ferritin concentration >700 μg/l or TSAT ≥40%) or low-dose i.v. iron, administered in a reactive fashion (0–400 mg monthly, if ferritin was <200 ng/ml or TSAT <20%). On completion, patients assigned to proactive high-dose i.v. iron experienced a reduction in the composite primary end point of nonfatal myocardial infarction, nonfatal stroke, hospitalization for HF, or all-cause death (hazard ratio [95% CI]: 0.85 [0.73–1.00]).^51^ In the proactive i.v. iron arm, the risk of the individual end points of myocardial infarction and hospitalization for HF was reduced by 31% (hazard ratio [95% CI]: 0.69 [0.52–0.93]) and 34% (hazard ratio [95% CI]: 0.66 [0.46–0.94]), respectively. Doses of ESAs were adjusted to maintain Hgb levels of 10.0 to 12.0 g/dl, but iron doses were not altered in response to changes in Hgb levels. Beyond an initial (~1 year) increase in Hgb that was more rapid in the group receiving proactive iron therapy, Hgb levels were similar between groups.^51^ A *post hoc* analysis revealed that the results were not completely explained by a reduction in ESA requirements, suggesting the benefits were conferred by iron repletion by nonerythropoietic mechanisms.^52^ Data from a recent retrospective study of an Italian cohort of patients on hemodialysis (*N* = 72) further support the notion that iron supplementation (i.e., i.v. ferric carboxymaltose [FCM]) reduces the risk of CV events.^53^

#### Correcting Anemia or ID in HF

Similar to data from CKD populations, the impact of Hgb normalization using darbepoetin alfa in patients with HF has been evaluated in the RED-HF (Reduction of Events by Darbepoetin Alfa in Heart Failure) clinical trial.^54^ Enrolled patients in RED-HF had HFrEF and anemia (Hgb level of 9.0–12.0 g/dl) without evidence of bleeding or other correctable causes of anemia. With a median eGFR of 45.7 ml/min per 1.73 m^2^, most patients met criteria for moderate-to-severe CKD. The median TSAT among the enrolled cohort was 24%. Targeting a Hgb level of 13.0 g/dl with darbepoetin alfa was associated with no discernable clinical impact on the risk of death from any cause or first hospitalization for worsening HF (hazard ratio [95% CI]: 1.01 [0.90–1.13]). Importantly, patients in the active treatment arm experienced significantly higher rates of thromboembolic events. On the basis of these findings, ESAs are not recommended in patients with HF. According to the 2017 guidelines of the American College of Cardiology, the American Heart Association, and the Heart Failure Society of America, ESAs should not be used to improve morbidity and mortality (a class III recommendation) and “cannot be recommended in patients with HF and anemia.”^18^ Similar recommendations against the use of ESAs are included in the European Society of Cardiology guidelines.^19^

An increasing body of evidence now supports the correction of ID in patients with HFrEF to improve patient outcomes. The benefits of correcting ID in patients with and without anemia with HF^55^^,^^56^ and the lack of efficacy supporting normalization of Hgb using ESAs in CKD and HF have led current HF management strategies to focus on correction of ID rather than raising Hgb levels. Specifically, i.v. FCM resulted in improvements in New York Heart Association functional class, 6-minute walk test performance, and quality of life measures in both the FAIR-HF (Ferinject Assessment in Patients with Iron Deficiency and Chronic Heart Failure) and CONFIRM-HF (Ferric Carboxymaltose Evaluation on Performance in Patients with Iron Deficiency in Combination With Chronic Heart Failure) trials.^56^^,^^57^ In both trials, ID was defined as ferritin <100 ng/ml or ferritin between 100 ng/ml and 300 μg/l if TSAT was <20%.^56^^,^^57^ In the 52-week CONFIRM-HF trial, the median total dose of FCM iron was 1500 mg (range: 500–3500 mg).^57^ Furthermore, i.v. iron resulted in improved exercise tolerance among patients with HFrEF and ID in the EFFECT-HF (Effect of Ferric Carboxymaltose on Exercise Capacity in Patients With Iron Deficiency and Chronic Heart Failure) study.^58^ In contrast, in the multicenter IRONOUT HF (Iron Repletion Effects on Oxygen Uptake in Heart Failure) trial, oral iron did not improve exercise capacity or quality of life among patients with HFrEF and ID.^59^ In a recently published study, however, oral sucrosomial iron was associated with improved exercise capacity and quality of life in patients with HFrEF and ID; however, only 25 patients were included in this trial, which was open label and not randomized.^60^ The current body of evidence, as described in the preceding texts, supports the use of i.v. iron to treat ID (serum ferritin <100 ng/ml or ferritin between 100 and 300 ng/ml and TSAT <20%) in HF with HFrEF regardless of the presence or absence of anemia to improve functional status and quality of life.^14^^,^^19^ The impact of iron replacement on patient outcomes in HF with preserved ejection fraction has not been reported, but the results of ongoing, randomized, placebo-controlled trials are expected soon.^61^

Although earlier trials of i.v. iron therapy have revealed significant improvements in quality of life and functional outcomes among patients with HF, they were not designed to evaluate the effects of treatment on event-driven or “hard” outcomes. More recently, evidence from meta-analyses^20^^,^^62^ and cohort studies,^63^ however, reveals significant reductions in the risk of HF hospitalization with i.v. iron therapy, independent of baseline Hgb levels.^20^^,^^63^ The early positive benefits have now been confirmed in the AFFIRM-AHF (A Randomized, Double-Blind, Placebo-Controlled Trial Comparing the Effect of Intravenous Ferric Carboxymaltose on Hospitalizations and Mortality in Iron-Deficient Subjects Admitted for Acute Heart Failure) trial.^21^ This multicenter, double-blind, randomized controlled trial included >1100 adults who were hospitalized for HF with concomitant ID. The i.v. iron, prescribed as FCM, was administered up to 4 times to correct ID (mean [SD] total dose of FCM administered during the study was 1352 [568] mg). The i.v. FCM reduced the risk of recurrent HF hospitalizations by 26% (rate ratio [95% CI]: 0.74 [0.58–0.94]) but did not affect the risk of CV mortality. In subgroup analyses of the primary end point (i.e., recurrent HF hospitalizations and CV mortality), the treatment effect of i.v. iron was numerically, but not significantly, greater in patients with baseline Hgb levels of ≥12.0 g/dl, suggesting the effects of therapy were not driven by improvements in anemia. In a meta-analysis of 4 randomized controlled trials (including AFFIRM-AHF), treatment with i.v. iron was associated with a 29% reduction in the risk of hospitalization for HF but did not affect all-cause or CV mortality.^64^

### Evidence for ID Correction in CKD: Data From HF Trials

With the exception of the PIVOTAL trial (conducted in patients receiving hemodialysis), there is scant evidence within the field of nephrology on the specific impact of ID correction alone on major outcomes separate from that of anemia correction. Although there is no substitute for well-designed, prospective randomized controlled trials to test this hypothesis, much can be learned from existing HF trials on the treatment effects of iron repletion within specific subgroups, such as those with CKD. Subgroup analyses (Table 2) from 3 clinical trials suggest that i.v. iron therapy may be beneficial in patients with CKD and coexisting HF. It is worth noting that none of these analyses evaluated more severe levels of kidney impairment separately from less severe stages of CKD. Although safety data for the CKD subgroups in these trials have not been reported separately, i.v. iron, as used in the setting of HF, has been generally well tolerated.^20^^,^^21^^,^^56^, ^57^, ^58^

**Table 2:** CKD subgroups in HF trials of i.v. iron

Study	Patient population	Treatment	CKD population	Results (overall cohort)	Results (CKD subgroups)
CONFIRM-HF^57^	HFrEF + ID (*N* = 304)	FCM vs. placebo	Mean (SD) eGFR: ~65 ml/min per 1.73 m^2^ eGFR <60 ml/min per 1.73 m^2^: ~35%	In the overall cohort, FCM was associated with a beneficial effect on 6MWT distance at week 24 (LS mean [SE] treatment effect: 33 [11] m)	i.v. iron was equally beneficial in patients with eGFR <60 and ≥60 ml/min per 1.73 m^2^ (predefined subgroup analysis)
FAIR-HF^56^	HFrEF + ID (*N* = 459)	FCM vs. placebo	Mean (SD) eGFR: ~64 ml/min per 1.73 m^2^ eGFR <60 ml/min per 1.73 m^2^: ~41%	In the overall cohort, FCM was associated with improvement based on the following: PGA (OR [95% CI]: 2.51 [1.75–3.61]); NYHA (OR for improvement by 1 class [95% CI]: 2.40 [1.55–3.71])	i.v. iron was equally beneficial in patients with eGFR <60 and ≥60 ml/min per 1.73 m^2^ (predefined subgroup analysis)
EFFECT-HF^58^	HFrEF + ID (*N* = 172)	FCM vs. placebo	Mean (SD) eGFR: ~52 ml/min per 1.73 m^2^	In the overall cohort, FCM was associated with a beneficial effect on peak oxygen consumption (LS mean [SE] treatment effect: 1.04 [0.44] ml/kg/min)	No CKD subgroup analysis available
Meta-analysis of 4 randomized controlled trials^20^	HFrEF + ID (*N* = 839)	FCM vs. placebo	Mean eGFR: ~63 ml/min per 1.73 m^2^ eGFR <60 ml/min per 1.73 m^2^: 44%	In the overall cohort, FCM was associated with reduced risk of: recurrent CV hospitalizations and CV mortality (RR [95% CI]: 0.59 [0.40–0.88]); recurrent HF hospitalizations and CV mortality (RR [95% CI]: 0.53 [0.33–0.86]); and recurrent CV hospitalizations and all-cause mortality (RR [95% CI]: 0.60 [0.41–0.88]	No CKD subgroup analysis available
AFFIRM-AHF^21^	Hemodynamically stable AHF (HFrEF) + ID (*N* = 1 108)	FCM vs. placebo	CKD as a documented comorbidity at baseline: 41% eGFR <60 ml/min per 1.73 m^2^: 52% eGFR tertile cutoffs: 42.96 and 64.32 ml/min per 1.73 m^2^	In the overall cohort, FCM was associated with RR (95% CI) of 0.79 (0.62–1.01) for the combined end point of total HF hospitalizations and CV death	i.v. iron was equally beneficial in patients across eGFR tertiles and across CKD status (i.e., present or absent) at baseline (predefined subgroup analysis)

6MWT, 6-minute walk test; AFFIRM-AHF, A Randomized, Double-Blind, Placebo-Controlled Trial Comparing the Effect of Intravenous Ferric Carboxymaltose on Hospitalizations and Mortality in Iron-Deficient Subjects Admitted for Acute Heart Failure; AHF, acute heart failure; CKD, chronic kidney disease; CONFIRM-HF, Ferric Carboxymaltose Evaluation on Performance in Patients with Iron Deficiency in Combination With Chronic Heart Failure; CV, cardiovascular; EFFECT-HF, Effect of Ferric Carboxymaltose on Exercise Capacity in Patients With Iron Deficiency and Chronic Heart Failure; eGFR, estimated glomerular filtration rate; FAIR-HF, Ferinject Assessment in Patients with Iron Deficiency and Chronic Heart Failure; FCM, ferric carboxymaltose; HF, heart failure; HFrEF, heart failure with reduced ejection fraction; ID, iron deficiency; LS, least squares; NYHA, New York Heart Association; OR, odds ratio; PGA, Patient Global Assessment; RR, rate ratio.

### Mechanistic Effects of Iron Therapy (Beyond Erythropoiesis)

As reviewed earlier, the physiological role of iron extends well beyond erythropoiesis. Because most of the body’s iron stores are contained in cells other than red blood cells,^23^ it seems likely that the favorable effects of iron repletion also transcend its beneficial impact on erythropoiesis. Given their considerable energy requirements—as evidenced by increased mitochondrial density—cardiac tissue and kidney tubular epithelium may be particularly responsive to iron therapy. In fact, cardiac magnetic resonance imaging sequences confirm the administration of i.v. iron to patients with HFrEF and ID results in repletion of iron in cardiac muscle and improvements in both right and left ventricular ejection fractions.^65^^,^^66^ Increases in myocardial iron content generally correlate with improvements observed in markers of HF severity (e.g., quality of life and functional capacity).^65^ In an *in vitro* model with human cardiomyocytes, iron repletion quickly corrected the mitochondrial dysfunction and the impaired contractility and relaxation associated with ID.^67^ The restoration of intracellular iron, after administration of i.v. iron, has also been associated with augmented skeletal muscle energetics among patients with HF, suggesting improved mitochondrial function.^68^ Finally, in the FAIR-HF trial, administration of i.v. iron in patients with HF and ID resulted in a modest increase in eGFR from baseline in 24 weeks.^69^ These improvements were independent of baseline eGFR (eGFR <60 and ≥60 ml/min per 1.73 m^2^) and Hgb levels (Hgb ≤12.0 and >12.0 g/dl) and are hypothesized to result from improved cellular energetics after iron repletion and/or improved renal hemodynamics.

Of course, the decision to replace iron stores with i.v. iron must also consider the safety of such therapy. Severe hypersensitivity reactions, although rare, occur at a rate of approximately 1 per 4167 persons exposed to nondextran formulations for the first time.^70^ Furthermore, i.v. iron does not increase the risk of infection.^71^, ^72^, ^73^ There is an increased risk of hypophosphatemia with some iron formulations, notably FCM and iron polymaltose.^74^ Other adverse events that can be associated with i.v. iron therapy include flushing, mild-to-moderate hypersensitivity reactions, and flu-like symptoms.^75^ Data suggest that current practices of iron administration do not pose a concern related to iron overload.^23^

### Conclusions

Current evidence and guidelines clearly support the use of i.v. iron in the setting of CKD anemia with concomitant ID. Despite such evidence-based recommendations, many patients, including those with Hgb levels <10.0 g/dl, remain undertreated.^76^ Moreover, there is a significant proportion of patients with CKD and ID who do not meet criteria for anemia and thus are frequently not treated. For these patients, evidence-based clinical recommendations are conspicuously lacking. Although there is a clear mechanistic rationale supporting the correction of ID in patients without anemia, clinical data supporting such an approach are largely absent (Figure 2). Data from several HF clinical trials suggest that treating “just” ID (i.e., independent of Hgb levels) is beneficial to patients with HFrEF. Because HFrEF cohorts generally include a substantial proportion of patients with CKD, the practice of correcting ID in patients with HFrEF and concomitant CKD seems safe and effective. Whether the clinical benefits of i.v. iron for ID extend to patients with CKD without HFrEF is untested and warrants additional study.

**Figure 2:**
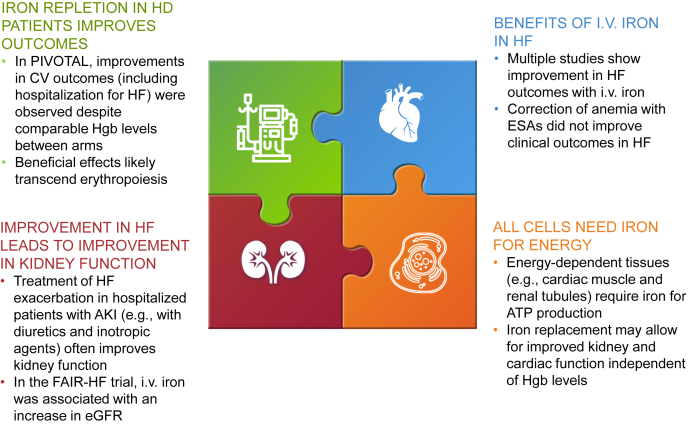
Treatment of ID in patients with CKD without anemia. AKI, acute kidney injury; ATP, adenosine triphosphate; CKD, chronic kidney disease; CV, cardiovascular; eGFR, estimated glomerular filtration rate; ESA, erythropoiesis-stimulating agent; FAIR-HF, Ferinject Assessment in Patients with Iron Deficiency and Chronic Heart Failure; HD, hemodialysis; HF, heart failure; Hgb, hemoglobin; ID, iron deficiency; PIVOTAL, Proactive Intravenous Iron Therapy in Haemodialysis Patients.

Although the introduction of hypoxia-inducible factor prolyl hydroxylase inhibitors may attenuate the requirements for supplemental iron in patients with anemia (secondary to increased iron availability), hypoxia-inducible factor prolyl hydroxylase inhibitors would not be expected to affect the treatment of populations without evidence of anemia. As such, we strongly recommend further mechanistic and clinical studies be conducted to explore the role of ID (rather than Hgb) as a therapeutic target in CKD. Until such data are available, it seems prudent to work with cardiologists to ensure correction of ID in patients with CKD with concomitant HFrEF and to make certain to follow evidence-based guidelines for repletion of iron stores in patients with CKD and evidence of anemia.

## Disclosure

JBW reports serving as a consultant to Akebia, AstraZeneca, Otsuka, Rockwell Medical, and Vifor Pharma and is on the speakers bureau for Akebia and AstraZeneca. SDA reports receiving grants from Abbott and Vifor International and personal fees from Abbott, Actimed, Bayer, Boehringer Ingelheim, Cardiac Dimensions, Impulse Dynamics, Novartis, Servier, and Vifor. JB reports serving as a consultant for Abbott, Adrenomed, Amgen, Applied Therapeutics, Array, AstraZeneca, Bayer, Berlin Cures, Boehringer Ingelheim, CVRx, G3 Pharmaceuticals, Impulse Dynamics, Innolife, Janssen, LivaNova, Luitpold, Medtronic, Merck, Novartis, Novo Nordisk, Relypsa, Sequana Medical, Vifor, and V-Wave Limited. AC reports receiving grants from AB-Biotics and Vifor Pharma; is or was a member of an advisory board for Astellas, AstraZeneca, GlaxoSmithKline, Novo Nordisk, and Vifor Pharma; and reports receiving speaker fees from Amgen, Arbor Research, Astellas, AstraZeneca, Bayer, and Vifor Pharma. AGS reports serving as a consultant to AstraZeneca, Grünenthal, Menarini, and Vifor Pharma. ICM reports receiving speaker fees, honoraria, and consultancy fees from Akebia, AMAG, Amgen, Astellas, Bayer, FibroGen, GlaxoSmithKline, Pharmacosmos, and Vifor Pharma.
